# Magnetic sentinel node biopsy with Sienna+®/SentiMag® in penile squamous cell carcinoma: Case report

**DOI:** 10.1016/j.eucr.2025.103227

**Published:** 2025-09-29

**Authors:** Diogo Carmali, Eduardo Felício, Sónia Afonso Ramos, António Pinheiro, Alberto Silva, Sara Duarte, Guilherme Bernardo, Filipe Gaboleiro, André Pita, Fernando Ferrito

**Affiliations:** Urology Department, Unidade Local de Saúde Amadora Sintra, Rua Teófilo Braga, 2720-276, Amadora, Portugal

**Keywords:** Penile cancer, Sentinel lymph node biopsy, SentiMag, Sienna+, Magnetic tracer

## Abstract

Penile squamous cell carcinoma (SCC) is a rare malignancy where accurate nodal staging is essential for prognosis and management. Dynamic sentinel lymph node biopsy with radiotracers is the standard approach but requires nuclear medicine facilities and exposes patients to radiation. We report a 62-year-old man with penile SCC who underwent glansectomy with sentinel lymph node biopsy using Sienna+®/SentiMag® and methylene blue. Two sentinel nodes were identified, one exclusively by Sienna+®/SentiMag®. Both were negative for metastasis. This case supports the feasibility of magnetic tracers as a radiation-free alternative in penile cancer staging.

## Highlights

• Accurate lymph node staging is crucial in penile squamous cell carcinoma.
• Sentinel lymph node biopsy with radiotracers is standard but involves radiation and logistics.
• Sienna+®/SentiMag® is a radiation-free alternative for sentinel node biopsy.
• In this case, one sentinel node was exclusively detected with the magnetic tracer.
• Magnetic sentinel node biopsy is feasible in penile cancer without nuclear medicine access.

## 1 Introduction

Penile squamous cell carcinoma (SCC) is a rare malignancy associated with significant morbidity.^1^ Accurate inguinal lymph node staging plays a critical role in establishing prognosis and guiding treatment. In patients with clinically node-negative disease (cN0) but high-risk pathological features (stage ≥ pT1b or grade ≥ G2), inguinal lymph node staging is indicated, with dynamic sentinel lymph node biopsy (SLNB) using radiotracers being the recommended approach.^2^ Sentinel lymph node biopsy using Sienna+®/SentiMag® (Sysmex, Milton Keynes, UK), a magnetic tracer system based on superparamagnetic iron oxide nanoparticles (SPIO), has emerged as a radiation-free alternative that may facilitate SLN detection, particularly in centers without access to nuclear medicine. We present a case of penile SCC treated with glansectomy and SLNB using both Sienna+®/SentiMag® and methylene blue.

## 2 Case presentation

A 62-year-old man with multiple comorbidities - including splenic marginal zone non-Hodgkin lymphoma under rituximab maintenance, Child-Pugh A liver disease, cerebrovascular disease with prior lacunar strokes, thrombocytopenia, arterial hypertension, dyslipidemia, obesity and presumed pulmonary silicosis - presented with a persistent whitish penile lesion first noted in September 2023.

Physical examination revealed an ulcerated leukoplastic lesion near the urethral meatus extending to the balano-preputial sulcus and ventral foreskin, with no palpable inguinal or femoral lymphadenopathies. An excisional biopsy with meatoplasty was performed in October 2024, showing a poorly differentiated (G3), HPV-negative invasive SCC. Staging computed tomography (CT) revealed no pelvic or inguinal lymphadenopathy.

Due to the tumor grade and clinically node-negative status (cN0), glansectomy with sentinel lymph node biopsy (SLNB) was planned.

**Fig. 1:**
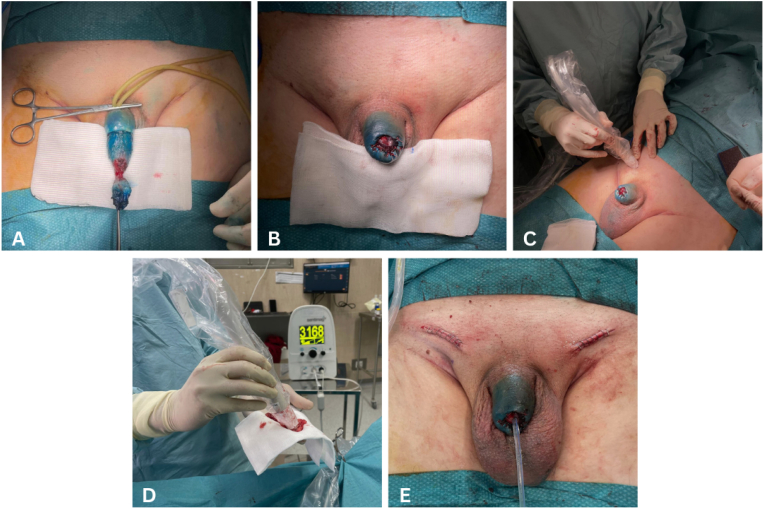
Intraoperative sequence of surgical procedure. A – Glansectomy; B – Glansectomy; C – Sentinel lymph node identification guided by the SentiMag® magnetometer after Sienna + injection; D – Confirmation of sentinel lymph node biopsy positivity (3168 units, above the ≥500-unit threshold for positivity; E − Surgical wound closure.

In March 2025, the patient underwent glansectomy preceded by perilesional injection of 2 mL of the **SPIO-based tracer Sienna+® at four quadrants around the lesion, administered 24 hours before surgery, according to manufacturer recommendations.** During the intervention, sentinel lymph node biopsy was guided by the SentiMag® magnetometer and methylene blue dye (Fig. 1). Two sentinel nodes were identified and excised: one in the right groin (SentiMag® signal 700 units, above the ≥500-unit **threshold for positivity,** methylene blue positive) and one in the left groin (SentiMag® signal 3168 units, also above threshold, methylene blue negative).

Histopathological examination confirmed a poorly differentiated penile SCC (pT1b) with perineural invasion, absent lymphovascular invasion, and negative surgical margins except for an involved deep margin. Both sentinel lymph nodes were free of metastatic involvement (pN0). Final pathological staging was pT1b pN0 R0 (margin-negative resection).

The patient recovered well in the immediate postoperative period and was discharged on postoperative day 3. However, three days later, he was readmitted with sepsis, presumed secondary to right orchitis, and was successfully treated with intravenous followed by oral antibiotics.

At one-month follow-up, the patient showed good recovery with satisfactory wound healing, catheter removal and no clinical evidence of recurrence or nodal disease.

## 3 Discussion

Sentinel lymph node biopsy is a valuable tool for staging of penile squamous cell carcinoma, particularly in patients with tumors beyond T1aG2 with clinically negative nodes (cN0). According to the European Association of Urology (EAU) guidelines, dynamic sentinel node biopsy (DSNB) using technetium-99m-labeled nanocolloids with or without methylene blue dye is the recommended technique for appropriate staging in intermediate- and high-risk patients. This method enables early micrometastasis detection while avoiding the morbidity associated with prophylactic bilateral inguinal lymphadenectomy.^2^

However, conventional DSNB requires access to nuclear medicine facilities, exposes patients and staff to ionizing radiation, and involves complex logistics, such as same-day radiotracer injection and surgery coordination.

The Sienna+®/SentiMag® system offers a promising radiation-free alternative to traditional DSNB. It uses a magnetic tracer (Sienna+®) composed of superparamagnetic iron oxide nanoparticles (SPIO) injected perilesionally up to 7 days before surgery. These particles travel via lymphatics to the sentinel lymph node, which can be localized intraoperatively using the handheld SentiMag® magnetometer.^3^^,^^4^

Advantages include simplified logistics, flexibility in scheduling, reduced patient contact time, elimination of radiation exposure and independence from nuclear medicine facilities. Additionally, it avoids the risks associated with methylene blue dye, such as permanent tattooing and rare anaphylactic reactions and does not appear to significantly prolong operative time, thereby improving surgical efficiency and overall patient experience.^5^ These characteristics may support the feasibility of broader implementation, even in low-volume or resource-limited centers.

Despite its benefits, the SentiMag® system is not without limitations. SPIO particles may cause prolonged pigmentation of lymph nodes and surrounding tissues, potentially complicating visual identification during surgery.^6^ Furthermore, residual iron particles can produce MRI artifacts lasting for months, which can interfere with postoperative imaging.^7^ Reported perioperative side effects of SPIO injection include mild discomfort or pain at the injection site, transient local inflammation, persistent pigmentation of regional tissues, and, very rarely, hypersensitivity reactions. None of these adverse effects were observed in our case. Another practical limitation observed in our case was the sensitivity of the magnetometer to nearby metallic surgical instruments. During the procedure, we noted occasional signal interference when standard stainless steel surgical instruments were used in close proximity to the magnetometer. Although this did not compromise node identification, it required more than one recalibration of the device. This limitation could potentially be minimized by employing non-metallic instruments, which could potentially simplify intraoperative workflow in future cases. Lastly, the initial setup cost and required training may also present barriers to widespread adoption.

The use of Sienna+ ®/SentiMag® is well validated in breast cancer and melanoma, with detection rates comparable to radioisotope methods.^8^, ^9^, ^10^, ^11^ However, its application in penile cancer remains limited. The SENTIPEN trial is the only prospective study so far, reporting equivalent sentinel node detection rates and safety, supporting Sienna+®/SentiMag® as a viable alternative in penile cancer management. Although promising, these results indicate that further optimization and validation are necessary before routine adoption.^5^

In our case, both magnetic and methylene blue tracers were used, allowing complementary detection. Notably, one sentinel node was identified only via Sienna+®/SentiMag®, highlighting its capacity for independent detection. This report adds to the limited but growing body of evidence supporting the feasibility of magnetic sentinel node biopsy in penile cancer, particularly in settings without access to nuclear medicine. Although the patient developed right-sided orchitis with sepsis during the postoperative course, this complication appeared unrelated to the use of the SentiMag® system or the SLNB technique.

## 4 Conclusion

This case demonstrates that SLNB using the Sienna+®/SentiMag® system is a feasible and well-tolerated approach for nodal staging in penile squamous cell carcinoma. The radiation-free technique offers logistical advantages and may serve as a viable alternative in centers lacking nuclear medicine. Although preliminary results are encouraging, further studies are necessary to validate its diagnostic performance and establish its role in routine clinical practice.

## CRediT authorship contribution statement

**Diogo Carmali:** Writing – original draft, Supervision, Resources, Methodology, Conceptualization. **Eduardo Felício:** Writing – review & editing, Investigation, Data curation. **Sónia Afonso Ramos:** Writing – review & editing, Supervision, Formal analysis, Conceptualization. **António Pinheiro:** Writing – review & editing, Investigation, Data curation. **Alberto Silva:** Writing – review & editing, Investigation, Funding acquisition, Data curation. **Sara Duarte:** Software, Resources, Methodology, Investigation. **Guilherme Bernardo:** Software, Resources, Methodology, Investigation. **Filipe Gaboleiro:** Software, Resources, Methodology, Investigation. **André Pita:** Software, Resources, Methodology, Investigation. **Fernando Ferrito:** Validation, Supervision, Project administration.

## Informed consent

Written informed consent was obtained from the patient for publication of this case report and any accompanying images.

## Ethics approval

Not applicable.

## Data availability statement

No datasets were generated or analyzed during the current study.

## Declaration of generative artificial intelligence (AI) and AI-assisted technologies in the writing process

The authors declare that no generative artificial intelligence (AI) or AI assisted technologies were used in the writing, editing or preparation of this manuscript.

## Funding

This study received funding from 10.13039/100017981Sysmex solely to cover publication fees and had no role in the study design, data collection, analysis, interpretation or decision to submit a case report for publication.

## Conflicts of interest

The authors declare no conflicts of interest.
